# The Effect of IL-17A and Combined Mechanical Injury on Meniscal Tissue Integrity In Vitro

**DOI:** 10.3390/jcm14217573

**Published:** 2025-10-25

**Authors:** Greta Ahrens, Florian Gellhaus, Jan-Tobias Weitkamp, Peter Behrendt, François Cossais, Bernd Rolauffs, Alan J. Grodzinsky, Bodo Kurz

**Affiliations:** 1 Department of Anatomy, CAU Kiel, Otto-Hahn-Platz 8, 24118 Kiel, Germany; greta.ahrens@web.de (G.A.);; 2 Clinic for Oral and Maxillofacial Surgery, UKSH Medical Center, Campus Kiel, Arnold-Heller-Straße 3, 24105 Kiel, Germany; 3 G.E.R.N. Research Center for Tissue Replacement, Regeneration & Neogenesis, Department of Orthopedics and Trauma Surgery, Faculty of Medicine, Medical Center, Albert-Ludwigs-University of Freiburg, 79085 Freiburg im Breisgau, Germany; 4 Department of Biological Engineering, Electrical Engineering and Computer Science and Mechanical Engineering, Massachusetts Institute of Technology, Cambridge, MA 02139, USA

**Keywords:** meniscus, IL-17, mechanical injury

## Abstract

**Objectives**: Meniscal integrity is crucial for knee joint stability and the prevention of osteoarthritis (OA) development. Recent studies suggested that mechanical overload and interleukin (IL)-17A may be important intertwined players in meniscal degeneration, but a direct impact of IL-17A on the meniscus has not been investigated. Therefore, the aim of this study was to analyze the effect of IL-17A on meniscal tissue with and without combined mechanical injury (MI). **Methods**: Meniscal explant disks (1 mm height, 3 mm diameter) were isolated from bovine menisci (preserving the native tibial superficial zone) and exposed to IL-17A [0–100 ng/mL] and/or MI (single compression, 50% strain, strain rate 1 mm/sec). After three days of incubation in a serum-free medium, the proteoglycan release (sGAG; DMMB assay), mRNA level of matrix-degrading enzymes (qRT-PCR), aggrecan degradation (NITEGE immunostaining), and cell death (histomorphometry of nuclear blebbing/apoptosis and condensed nuclei/unspecified cell death) were determined. Statistics: one- and two-way ANOVA with Tukey’s multiple comparisons or Kruskal–Wallis with post hoc testing. **Results**: IL-17A increased sGAG release in a dose-dependent significant manner. MI also induced the release of sGAG significantly, but the combination with IL-17A showed the highest levels. Both IL-17A and MI individually affected the mRNA levels for ADAMTS4 and MMP-13 slightly, but the combination of both particularly induced a significant increase in mRNA levels. Signals for the ADAMTS4-related aggrecan neoepitope NITEGE were elevated by IL-17A in superficial areas of the excised tissue and by MI in superficial and deeper areas. The combination of both stimuli intensified this signal further. MI increased the number of cells with condensed nuclei significantly and induced apoptosis in a small proportion of cells. IL-17A had no significant impact on the amount of condensed or apoptotic nuclei. **Conclusions**: Our findings emphasize an interaction between inflammatory cytokine IL-17A signaling and mechanical stress since IL-17A induced matrix degeneration in meniscal tissue, which intensified in combination with a trauma. The latter might create a post-traumatic environment that promotes meniscal degeneration and subsequently osteoarthritis progression.

## 1. Introduction

Osteoarthritis (OA) is one of the most common age-related diseases worldwide [1], and up to now, the therapeutic endpoint is often an artificial joint replacement [2]. In addition to limitations in the functional joint movement and related pain of the patient, OA is a major health-economic challenge [3]. OA is associated with pro-inflammatory processes in general [4] and therefore, inflammation is a central event involving the production of various cytokines, such as tumor necrosis factor-α (TNF-α) and interleukin (IL)-1. These are known to be elevated in OA patients and contribute to extracellular matrix (ECM) degradation and related enzyme synthesis [5]. OA can also be the result of chronic joint overloading or incorrect loading, which in the course of time leads to the destruction of the cartilage and meniscal tissue and, finally, the whole joint [6]. OA can be triggered by an acute cartilage or meniscus injury, in the sense of post-traumatic OA (PTOA) [7], and through the loss of meniscal integrity, be it through injury or meniscectomy [8]. Wang et al. (2020) recently investigated the underlying mechanism of partial anterior cruciate ligament injuries leading to meniscus degeneration in a rabbit model and concluded for the first time that elevated levels of the pro-inflammatory cytokine IL-17, along with increased matrix metalloproteinase (MMP)-13 expression, contribute to mechanically induced meniscus degradation [9]. This highlights the combined importance of mechanical stress and IL-17 in that context; however, the direct impact of IL-17 on meniscal tissue remains unknown so far.

Indeed, IL-17A has been measured in the synovial fluid of OA patients and in a cross-sectional study with primary knee OA patients [10,11]. Elevated IL-17A levels were significantly linked to longer disease duration, increased pain scores, poorer quality of life, severe disability, and advanced structural damage. It is proposed that therapeutics targeting IL-17A should be further investigated [11,12,13]. However, to achieve this, it is crucial to comprehend how and under what conditions IL-17A initiates joint tissue degeneration. The destructive effect of IL-17A on articular cartilage causing an increase in GAG release and matrix degenerating enzyme activity has recently been demonstrated [14].

The interleukin-17 (IL-17) cytokine family consists of six members, designated IL-17A through IL-17F, which share structural homology but exert distinct biological functions. IL-17A, the prototypical and most extensively studied member, is mainly produced by Th17 cells but also by γδ T cells, innate lymphoid cells, NK cells, and mast cells. It forms homodimers (IL-17A/A) or heterodimers with IL-17F (IL-17A/F), signaling primarily through a heterodimeric receptor complex composed of IL-17RA and IL-17RC. IL-17F, which shares approximately 55% sequence identity with IL-17A, is also expressed by Th17 and innate immune cells, but exerts weaker biological activity. Both IL-17A and IL-17F have been implicated in the pathogenesis of inflammatory joint diseases such as osteoarthritis and rheumatoid arthritis, where increased concentrations have been detected in synovial fluid, meniscal tissue, and articular cartilage, with IL-17A levels in osteoarthritic synovia typically ranging from 5 to 10 pg/mL [11,13,15].

Other IL-17 family members display more specialized expression profiles. IL-17B is expressed in various tissues, including endocrine organs, and signals through IL-17RB. IL-17C, predominantly produced by epithelial cells, signals via an IL-17RA/IL-17RE complex and has been linked to barrier integrity and mucosal defense. IL-17D is the least understood member, expressed in non-immune tissues such as the heart, adipose tissue, and the brain; its receptor is not fully defined, although CD93 has been suggested as a candidate. IL-17E (also known as IL-25) is structurally more divergent and functions through IL-17RA/IL-17RB to promote type-2 immune responses, including eosinophilia and allergic inflammation. The IL-17 receptor family comprises five known subunits (IL-17RA–RE), all of which are type-I transmembrane proteins with extracellular fibronectin-III-like domains and a conserved intracellular SEFIR domain. IL-17RA is ubiquitously expressed and participates in most IL-17 receptor complexes, underlining its central role in IL-17 signaling. Signal transduction requires the adaptor protein ACT1, which mediates the downstream activation of NF-κB, MAPK, and C/EBP pathways, thereby promoting the expression of pro-inflammatory mediators. Within the joint environment, IL-17A and IL-17F are of particular relevance. Elevated levels of these cytokines have been found in the synovial fluid of patients with osteoarthritis and rheumatoid arthritis compared with healthy controls, and IL-17 has been shown to induce catabolic processes in articular cartilage [16,17].

Several studies have suggested that there might be interactive and potentially additive or even synergistic effects of cytokines in combination with mechanical stimulation or injury. For example, IL-1α and TNFα can cause a synergistic loss of proteoglycans from mechanically injured bovine and human cartilage [17,18]. Mechanical injury (MI) also potentiates the catabolic effects of TNFα and IL-6/sIL-6R in causing proteoglycan degradation in human and bovine cartilage [19]. Since levels of IL-17A in OA joints are elevated but still relatively low, it might be of interest whether effects triggered by that cytokine can be influenced by MI too. Therefore, the aim of this study was to investigate for the first time the direct impact of IL-17A on meniscal tissue alone and in combination with MI. It is hypothesized that IL-17A and MI might cause synergistic meniscus destruction. These results could help to identify IL-17A as a potential therapeutic target in the treatment of meniscus-related OA or PTOA.

## 2. Materials and Methods

### 2.1. Isolation and Culture of Meniscal Explants

Meniscal explants (n = 1144) were harvested from 18 donor animals and subsequently maintained in culture.

**Figure 1:**
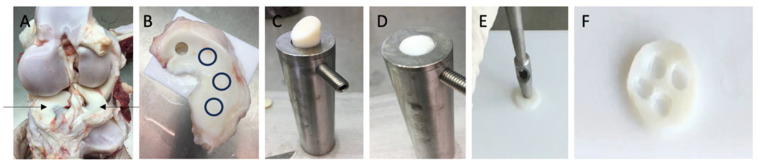
Description of the explant harvesting procedure: (**A**): depiction of the opened bovine knee joint highlighting medial and lateral menisci (indicated by arrows); (**B**): excised meniscus with one 1 cm cylinder already punched; remaining cylinder harvest sites are indicated by black circles; (**C**): placement of the cylinder in the custom sampler holder (diameter 1 cm); (**D**): trimmed cylinder (1 mm height, 1 cm diameter) while containing the native tibial superficial zone; (**E**): process of preparing 3 mm explants from the 1 cm disk using a punch, (**F**): 1 cm disk after harvesting the 3 × 1 mm explants.

Knee joints were obtained from cattle aged 18–24 months that had been slaughtered the previous day and stored under refrigerated conditions. Within 20 h post-mortem, intact bovine knee joints were aseptically opened under a laminar flow hood using a scalpel. Both medial and lateral menisci were carefully excised by severing their bony and capsular attachments (Figure 1A). The isolated menisci were subsequently washed in phosphate-buffered saline (PBS) supplemented with 10% penicillin G (10,000 U/mL) and streptomycin sulfate (25 µg/mL; P/S).

Full-thickness cylindrical explants (10 mm in diameter) were punched from the central circumferential region of the menisci using a 1 cm biopsy punch (see Figure 1B). Cylinders were then transferred to a custom cutting apparatus featuring a 1 mm deep groove and a 1 cm diameter guide (see Figure 1C). Using a scalpel, the cylinders were trimmed along the guide to a thickness of 1 mm while preserving the native tibial superficial zone (see Figure 1D). The resulting meniscal disks were subjected to an additional washing step.

Each disk yielded four smaller explants (3 mm in diameter, 1 mm thick), prepared with a 3 mm biopsy punch. (Hebu-medical, Tuttlingen, Germany) (see Figure 1E,F) and washed three additional times. Minor deviations in explant thickness were observed due to the preparation process; therefore, all explants were individually measured with a digital caliper (mean height of 1.195 ± 0.005 mm; n = 1144 explants). Explants were randomly allocated individually into 96-well plates and cultured individually in 250 μL of medium per well. Following a 24 h equilibration phase at 37 °C in a humidified atmosphere of 5% CO_2_, cultures were maintained in low-glucose Dulbecco’s modified Eagle’s medium (DMEM; Biochrom, Berlin, Germany) supplemented with 10 mM HEPES buffer (Biochrom), 0.4 mM proline (Sigma-Aldrich, Darmstadt, Germany), 1 mM sodium pyruvate (Biochrom), 50 μg of vitamin C, 100 U/mL of penicillin G, 100 mg/mL of streptomycin, 0.25 mg/mL of amphotericin B (PAA Laboratories, Pasching, Germany), 0.1 mM non-essential amino acids, and 1% ITS liquid media supplement (Sigma-Aldrich). Low-glucose DMEM was used to maintain a physiological culture environment and to preserve chondrocyte phenotype during expansion, as previously demonstrated by Heywood et al., who reported enhanced chondrogenic capacity of cells cultured under low-glucose conditions [20].

The use of low-glucose DMEM was further justified by the aim of enhancing the comparability of the results, given that this medium composition had been previously established and standardized within our research group [21].

### 2.2. Incubation and Cytokine Stimulation with IL-17A

After the equilibration phase, treatment commenced by renewing the culture medium, either supplemented with bovine IL-17A or maintained without cytokine addition (Kingfisher Biotech, Saint-Paul, MN, USA) in different concentrations [10 pg/mL–100 ng/mL] and performed for 72 h at the same culture conditions. After that, supernatants were frozen, and explants were collected for real-time qPCR or histological analysis.

### 2.3. Mechanical Injury (MI)

MI was applied by a single load compression after the equilibration phase using an incubator-housed computer-controlled loading device and a protocol already established in the study group [22]. In brief, a controlled displacement to 50% strain was applied to the individual explants, using the original explant cutting thickness as the starting point; stamp velocity was 1 mm/s. The compression was held for 10 s and then leveled back to the starting position. Stamp displacement and compressive load were measured during compression.

### 2.4. Histological Detection of Cell Death and Immunohistochemical Analysis of Aggrecan Degradation

To assess apoptotic cell death, characterized by condensed nuclei or nuclear blebbing [23], explants were fixed overnight in 4% paraformaldehyde (PBS), embedded in Paraplast (Carl Roth GmbH, Karlsruhe, Germany), sectioned at 7 μm, and subsequently stained with Mayer’s hematoxylin, following established procedures [24]. Apoptotic cells, identified by nuclear condensation or morphological hallmarks of apoptosis, were quantified as a percentage of the total number of cells per optical field by a blinded investigator. Data are based on three independent experiments. In each experiment, five different explants per experimental group had been sectioned, and the cell values for each section were calculated from three different section areas (fields of vision). Since cutting of the explants induces apoptosis at the edges of the tissue, the margins of the sections (~150 µm thickness) were excluded. Images were taken using a Zeiss Axiophot microscope (Zeiss, Wetzlar, Germany).

Aggrecan cleavage was exemplary, shown by staining of the cleavage product NITEGE. For immunohistochemical visualization of aggrecanase-specific aggrecan neoepitope NITEGE formation, histological sections were incubated for 2.5 min in a digester at 100 °C (in 0.01 M citric acid, pH 6.0) and thereafter incubated overnight at 4 °C with the primary antibody (Rabbit-IgG-Aggrecan Neo Polyclonal Antibody PA-1746; 1:50 dilution in 1% BSA; Thermofisher Scientific, Waltham, MA, USA). Sections were rinsed in Tris-NaCl and incubated with the secondary antibody AlexaFluor 488 goat anti-rabbit IgG (1:700 dilution; Invitrogen, Carlsbad, CA, USA) for one hour at room temperature. For supplementary nuclear visualization, explants were stained with bisbenzimide (Sigma-Aldrich, St. Louis, MO, USA), and images were obtained using a ZEISS Apotome fluorescence microscope (Jena, Germany). NITEGE staining was performed solely to provide a representative visualization of proteoglycan degradation induced by different stimuli.

### 2.5. Measurement of Glycosaminoglycan (GAG) Release

Glycosaminoglycan (GAG) release from explants into the supernatant was determined via the dimethylmethylene blue (DMMB) assay at 525 nm (Ultraspec II photometer, Biochrom, Cambridge, UK), employing shark chondroitin sulfate as a reference standard. Data are presented as μg GAG per ml, normalized to explant height (mm) using a cell culture model previously established within our research group [21]. In addition to IL-17A, IL-1ß (R&D Systems, Inc., Abingdon, UK; 10 ng/mL) served as a positive control group that was already proven to show significant results [25].

### 2.6. Quantitative Real-Time PCR (qPCR)

Relative quantification of target gene transcription was carried out using quantitative real-time PCR (qRT-PCR). For gene expression analysis, meniscal explants were pooled to obtain sufficient RNA yield per biological replicate, since meniscus did not contain a very high cell density. From each donor animal, both menisci of one knee joint were excised and processed as described above to generate explants measuring 1 × 3 mm. Treatments were performed individually in well plates under identical culture conditions. After completion of the incubation period, eight explants belonging to the same experimental group were pooled to form one sample for RNA extraction. Each donor animal represented one independent biological replicate. After 72 h of treatment, the pooled explants were immediately frozen in liquid nitrogen. The manufacturer of the TRIZOL reagent recommends using 100 mg of tissue for an isolation. Due to the low cell density in meniscus tissue, we used 150 mg. Total RNA was extracted after pulverization (Silent Crusher S, Heidolph, Germany) of the tissue using the TRIZOL reagent (1 mL/150 mg wet weight tissue; Invitrogen, Carlsbad, CA, USA). Extraction was performed according to the manufacturer’s instructions. In brief, the tissue was pulverized and centrifugated to remove tissue and cell fragments and homogenized with the TRIZOL reagent in multiple repetitive steps (phase separation). Aquagenus phase (containing the RNA) was then taken and filled with isopropyl up to 1 ml. Again, it was centrifuged to form a RNA pellet. The supernatant was discarded and washed with ethanol twice. Again it underwent centrifugation, with the supernatant discarded, and the RNA was dried under open air. Finally, the RNA pellet was redissolved in aqua. RNA purity was controlled by measuring OD_260_/OD_280_ ratio (Take 3 Epoch BioTek, Agilent, Santa Clara, CA, USA). RNA concentration was taken. Remaining DNA contamination was removed by a DNase digestion kit by manufacturer’s instructions (65 °C for 10 min; RQ1 RNase-Free DNase; Promega, Madison, WI, USA). Following this, the RNA was isolated and overwritten into a cDNA using Revert Aid H Minus Reverse Transcriptase (Thermofisher Scientific, Waltham, MA, USA). This process was carried out according to the manufacturer’s instructions. Based on the previously measured RNA concentration, the necessary volume for 400 ng of RNA was added (total volume = 18 µL, 11 µL from DNase digestion including the 400 ng RNA, 4 µL 5× RT buffer, 1 µL DNTPs (all 10 mM), 1 µL randomized hexamer primer (100 µM, 1 µL reverse transcriptase (200 U/µL))). According to the manufacturer, incubation was carried out for 75 min in the following steps: 25 °C for 10 min, 42 °C for 50 min, 70 °C for 15 min.

Quantitative real-time PCR (qPCR) was carried out immediately on a 7500 Fast Real-Time PCR System (Applied Biosystems, Darmstadt, Germany) using the QuantiTect SYBR^®^ Green PCR Kit (Qiagen, Hilden, Germany) in accordance with the manufacturer’s guidelines.

Bovine primer sequences are shown in Table 1 (Merk Sigma-Aldrich, Darmstadt, Germany). Targets for the primers are GAPDH (G3PDH, sequence accession NM_001034034.2, intron-spanning, Amplicon length 101 bp) [26], MMP-13 (sequence accession NM_174389.2, only exon-spanning, Amplicon length 101 bp) [27], and ADAMTS4 (sequence accession NM_181667.1, intron-spanning, Amplicon length 100 bp [26]). Primer sequences are used as previously described [26]. In general, if genomic DNA is removed during RNA extraction or during reverse transcription, and only RNA remains, then designing primers that do not span introns typically has little impact. However, if gDNA is not removed during RNA extraction, the resulting RNA sample will contain residual genomic DNA. In such cases, if the primers do not span introns, the primers may amplify DNA fragments in addition to cDNA, leading to falsely elevated expression levels of mRNA. In this study, this process is of no relevance since a DNAse model was used in RNA expression. “Exon-spanning” in gene primers means that the primers are designed to span two adjacent exons and encompass an intervening intron. This approach is often used in PCR (polymerase chain reaction) to ensure that only genomic DNA (gDNA) is amplified and not contaminants from mRNA. Because mRNA has already excised the introns, exon-spanning primers cannot generate products from pure RNA samples, making them a useful tool for quality control and distinguishing gDNA from mRNA. The primers were predesigned from the manufacturer [28,29].

As previously described [27], a special 96-well plate (Sarstedt, Nümbrecht, Germany) was used for the qPCR analysis. Each well included a total volume of 25 µL consisting of SYBR Green I Reagent 12.5 µL, 0.3 µL of Primers (Sense and Antisense, each 0.5 µM as recommended by the instructor; for details see Table 1), 11.9 µL of Aqua dest., including 40 ng of cDNA. Each analysis was performed in duplicate. The SYBR Green I solution also included the HotStar Taq-DNA-polymerase, dNTPs, as well as 2.2 mM MgCl_2_ according to the manufacturer. Cycler protocol: one cycle with 78° for 15 min, followed by 40 cycles of 94° for 15 s, 60° for 30 s, and 72° for 30 s. All ct values for the NTC were >40 cycles. All samples were assayed in duplicate, and relative quantification was performed using the ΔΔCT approach. Expression of the gene of interest was first normalized to the reference gene GAPDH (ΔCT) and subsequently to the untreated control group (ΔΔCT). Fold changes in target gene expression were calculated using the Livak method (2^−ΔΔCT^) [30]. GAPDH was chosen as housekeeping gene based on previous studies; mean ct values were around 30.46 (SEM 0.1787) [31,32,33,34,35].

### 2.7. Statistical Analysis and Power Analysis

A total of 1144 meniscal explants were collected from 18 donor animals. From each animal, explants were harvested from both the medial and lateral menisci of the knee joint. Following harvest, explants were randomly allocated, individually isolated, and measured. Each donor animal constituted a single independent experiment (n), ensuring that biological variability between animals was captured in the study design.

Each independent animal was defined as one biological replicate (n = 1). This n also represents independent experiments, as measurements were performed at different time points. The statistical analysis was not based on the mean values of individual experiments but was performed using the technical replicates (i.e., different explants obtained from the same donor animal) as the statistical unit. For the analysis of GAG loss, explants from three independent donor animals were used in three individual experiments. Each experimental group included five technical replicates, resulting in a total of 15 data points used for statistical analysis. Apoptosis quantification was likewise based on three independent experiments, each comprising five technical replicates per group. For the qPCR analysis, as described in the Section 2.6, eight explants per experimental group were pooled for RNA extraction and subsequent PCR. In total, eight independent experiments were conducted, corresponding to eight donor animals (n = 8). For the NITEGE immunofluorescence analysis, a qualitative assessment was performed using samples from three independent experiments, corresponding to three donor animals.

All datasets were evaluated for normality using the Kolmogorov–Smirnov test. Statistical analyses were conducted in GraphPad Prism 10 (GraphPad Software Inc., San Diego, CA, USA). Group comparisons were performed using one- or two-way ANOVA with Tukey’s multiple comparisons test for parametric data and the Kruskal–Wallis test with Dunn’s post hoc analysis for non-parametric datasets. Partly, a test for trends had been performed (based on ANOVA). If data allowed (single value for each group, all from one animal), they had been matched/paired. Differences were considered significant if *p*  ≤  0.05. Quantitative data in the text are presented as mean and standard error of the mean (SEM). Partly, a regression analysis with a three-parameter test was conducted and the best-fitting model was selected as indicated in the results. All graphs present pooled data from repeated experiments. An a priori power analysis has been performed using G*Power (Version 3.1). The dose-dependent effect of GAG release was calculated to use n = 2 biological replicates with each 7 groups based off previously performed research to achieve an actual power of 0.98. For qPCR based on previous published data, n = 5 was determined to achieve a power of 0.95. For the counting of nuclear blebbing, n = 3 was determined to achieve a power of 0.99 based on previously published data.

## 3. Results

### 3.1. Effect of IL-17A on the Glycosaminoglycan (sGAG) Release in Meniscal Tissue

**Figure 2:**
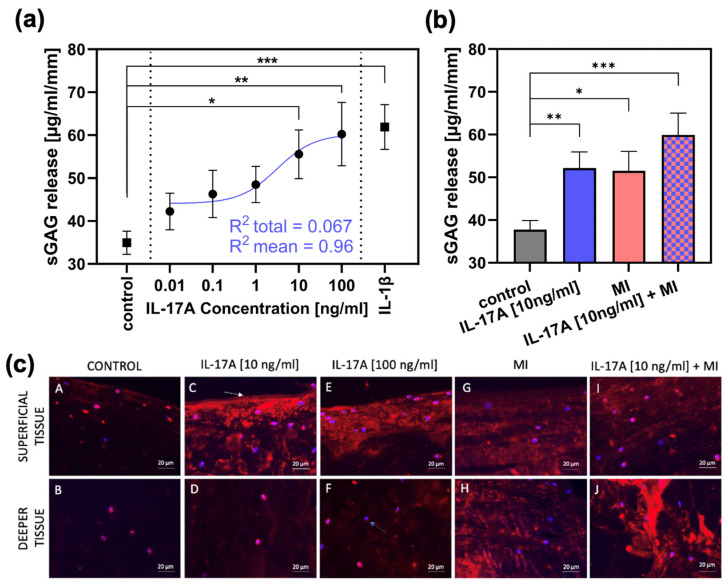
The effect of IL-17A and mechanical injury (MI) on proteoglycan degradation in meniscal tissue after 3 days of cultivation. (**a**) sGAG release (µg/mL/mm) from meniscal tissue treated with different concentrations of IL-17A or 10 ng/mL IL-1β, tested against the control group (n = 3). Additionally, a regression has been performed. The shown non-linear model was best fitting, with a R^2^ = 0.067 towards the total amount of values and a R^2^ = 0.96 to the mean values. Statistical significance was determined using Dunn’s multiple comparison test. (**b**) sGAG release (µg/mL/mm) after MI and treatment with 10 ng/mL IL-17A (control, IL-17A n = 6; MI, IL-17A + MI n = 3). All values are given as means ± standard error of the mean (SEM); * indicates experimental groups that are significantly different from the control group (* = *p* < 0.05, ** = *p* < 0.01 *** = *p* < 0.001); statistical significance was determined using Tukey’s multiple comparison test. (**c**) Figures A to J show aggrecan neoepitope (NITEGE) distribution generated by aggrecanase cleavage (shown by red staining); blue bisbenzimide staining = nuclei (blue arrow(F)); white arrow (C): original surface of the bovine meniscal tissue. Bar = 20 µm.

Release of sGAG from meniscal explants (normalized to explant height and measured after three days of cultivation, n = 4) was 34.95 ± 2.7 µg/mL/mm (control group; Figure 2a). Stimulation with increasing dosage of interleukin-17A [0.01 ng/mL-100 ng/mL; in steps of a tenfold multiplication] resulted in elevated sGAG levels in the supernatant reaching significance compared towards the control group from 10 ng IL-17A/mL onwards with a release of 55.56 ± 5.7 µg/mL/mm (*p* = 0.0217) and a release of 60.24 ± 7.4 µg/mL/mm (*p* = 0.0075) for 100 ng IL-17A/mL. For internal validation, the treatment with 10 ng/mL interleukin-1β as a classical pre-inflammatory reference cytokine resulted in a significant increase in sGAG release up to 61.89 ± 5.2 µg/mL/mm (compared with control; *p* = 0.0003). Additionally, regression has been performed and the best-fitting line is shown in Figure 2a (blue). This regression achieved a R^2^ = 0.067 towards the total amount of values and a R^2^ = 0.96 towards the mean values.

### 3.2. Effect of IL-17A in Combination with Mechanical Injury (MI) on Proteoglycan Degradation

In an extra set of experiments, an IL-17A treatment of 10 ng/mL was combined with a single injurious mechanical compression (MI) prior to the three-day cultivation (n = 3). The MI induced an average peak stress of 18.09 ± 5.97 MPa in the meniscal tissue. After three days, the control group showed a sGAG release of 37.8 ± 2.14 µg/mL/mm (Figure 2b), which was increased significantly by IL-17A (10 ng/mL) to 52.1 ± 3.82 µg/mL/mm (*p* = 0.0043) and by MI to 51.51 ± 4.54 (*p* = 0.02). However, the combination of the two stimuli resulted in the highest release in sGAG to 59.94 ± 5.08 µg/mL/mm (*p* = 0.0007).

Degradation of aggrecan was visualized by immunohistochemical staining of the aggrecan neoepitope NITEGE (Figure 2c). The control group (Figure 2c, picture A,B) showed very low signals, but treatment with IL-17A in a concentration of 10 ng/mL caused a signal increase, especially in the superficial tissue areas (Figure 2c, picture C) of the meniscal explants. Increasing the dose to 100 ng IL-17A/mL resulted in a subtle increase in signal intensity even in the deeper tissue (Figure 2c, picture F). MI resulted in consistent NITEGE-signal enhancement in both the superficial (Figure 2c, picture G) and deep layers (Figure 2c, picture H). The combination of MI and IL-17A induced a signal increase especially in the deep zones (Figure 2c, picture J), showing the highest signal intensity compared with all other test groups and layers.

**Figure 3:**
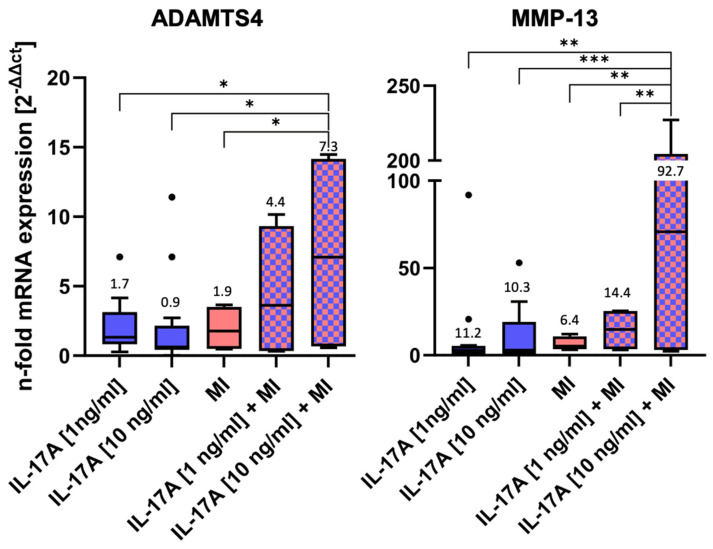
mRNA levels of matrix-degrading enzymes after 3 days of incubation ± interleukin-17A or mechanical injury (MI) using the ΔΔCT method for calculation (control level = 1). For each experimental group, eight randomized explants per animal were pooled for mRNA isolation and 8 independent experiments were performed (n = 8). Stars indicate experimental groups that are significantly different from each other (* = *p* < 0.05, ** = *p* < 0.01 and *** = *p* < 0.001). Statistical significance was determined using Tukey’s multiple comparison test. All values in the figure are given as Box and Whisker (Box: Median + Interquartile range; bar: Q1/Q3 ± 1.5 × Interquartile range; outliners: dots ± standard error of the mean (SEM)).

MRNA levels of two of the major matrix-degrading enzymes, namely ADAMTS4 and MMP-13, were measured after 3 days of incubation by qPCR (Figure 3).

Both IL-17A and MI induced little, but non-significant, alterations in mRNA levels compared WITH the normalized control (control = 1, not shown in the graph) (Figure 3): Stimulation with IL 17A alone did not result in a dose-dependent difference in the RNA expression of ADAMTS4 and MMP13 between 1 ng and 10 ng. Nevertheless, a dose-dependent effect was observed when IL 17A was combined with mechanical injury. IL-17A affected ADAMTS4 levels by a factor of 1.7 ± 0.4 [1 ng/mL] (*p* = 0.76) or 0.9 ± 0.2 [10 ng/mL] (*p* = 0.75), respectively, and MI ADAMTS4 levels by a factor of 1.9 ± 0.8 (*p* > 0.99). However, the highest ADAMTS4 mRNA levels were found in the combined IL-17A + MI treatment groups. Compared with the control, levels increased 4.4-fold ± 2.4 for 1 ng IL-17A/mL (*p* = 0.4) and, significantly, 7.3-fold ± 3.8 for 10 ng/mL (*p* = 0.002). Also, the values after the combined treatment with 10 ng IL-17A + MI were found to be significantly higher than in the groups with individual treatments (versus MI alone *p* = 0.011, versus IL-17A alone *p* = 0.0221).

Comparable effects could be observed for MMP-13 mRNA levels (Figure 3). The application of 1 ng IL-17A /mL caused a slight but non-significant increase by a factor of 11.2 ± 7.5 (*p* = 0.94) or 10.3± 4.2 for 10 ng IL-17A/mL (*p* = 0.95). The mechanical injurious compression led to a non-significant 6.4-fold ± 2.0 increase (*p* > 0.99). However, a combination of the stimuli dose-dependently increased MMP-13 mRNA levels 14.4-fold ± 6.2 for 1 ng IL-17A /mL + MI (*p* = 0.99) and, significantly, 92.7-fold ± 54.6 for 10 ng IL-17A /mL + MI (*p* = 0.0002 vs. control). Comparable to the ADAMTS4 results, the combination of 10 ng IL-17A + MI increased MMP-13 levels significantly in comparison with the other experimental groups: *p* = 0.0013 vs. 1 ng IL-17A/mL, *p* = 0.0009 vs. 10 ng IL-17A/mL, *p* = 0.0024 versus MI alone, and *p* = 0.007 vs. 1 ng IL-17A/mL + MI).

### 3.3. Effect of IL-17A in Combination with Mechanical Injury on Cell Death

**Figure 4:**
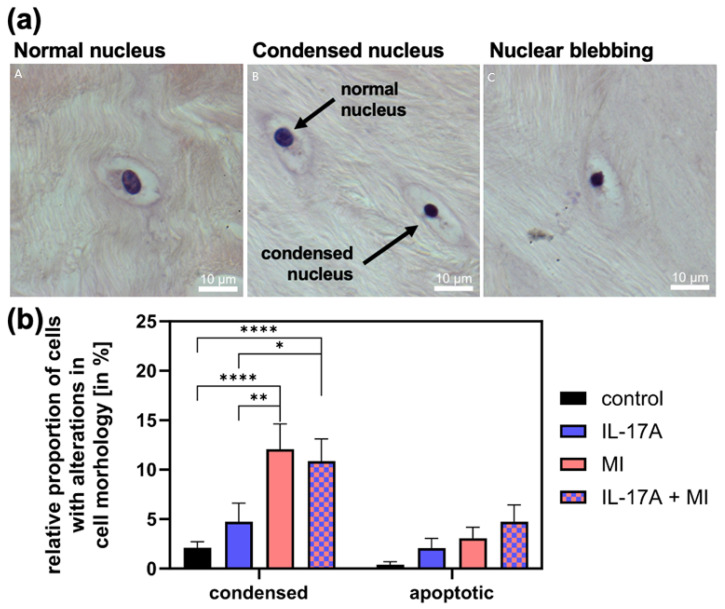
(**a**) Examples of cells with normal (A), condensed (B) and apoptotic nuclei (C). (**b**) Relative proportion of apoptotic cells with nuclear blebbing and cells with a condensed nucleus of the total cells in the meniscal tissue 72 h after mechanical injury (MI) and/or treatment with 10 ng/mL interleukin-17A (IL-17A). Histomorphological analysis of hemalaun-stained paraffin sections. N = 3 independent experiments with a total of 15 explants for each experimental group. * Indicate experimental groups that are significantly different from each other with * = *p* < 0.05, ** = *p* < 0.01, **** = *p* < 0.0001; statistical significance was determined using Tukey’s multiple comparison test. All values in the figure are given as means and standard error of the mean (SEM).

To assess changes in cell viability-related morphology, we counted cells with condensed nuclei and apoptotic cells (exhibiting nuclear blebbing, see Figure 4a for examples) and normalized these to the total cell count. The proportion of condensed cell nuclei in meniscal tissue increased significantly due to MI from 1.6 ± 0.5% (controls) to 11.6 ± 4% (*p* < 0.0001) and that of apoptotic cells (control: 0.0 ± 0.0%) non-significantly to 2.6% ± 0.3 (*p* = 0.51; see Figure 4b). With the addition of IL-17A [10 ng/mL], the amount of condensed cell nuclei was comparable to the control group with 1.7 ± 1.0% (*p* = 0.53) and numbers of apoptotic cell nuclei increased slightly to 1.6 ± 0.5% (*p* = 0.82). The combination of IL-17A and MI increased the number of condensed nuclei significantly compared with the control to 11.0 ± 2.7% (*p* < 0.0001) (Figure 4b) and also compared with the supplement of IL-17A alone (*p* = 0.025) but showed no increase from MI alone (*p* = 0.94). The combination of IL-17A and MI caused an additive increase in apoptotic cells to 4.0 ± 1.4%, which, however, failed to be significant under the given circumstances (*p* = 0.11) but showed a linear trend of increasing apoptosis, in the shown order in Figure 4b (*p* = 0.0016; R^2^ = 0.993).

## 4. Discussion

In the present work, the influence of the pro-inflammatory cytokine IL-17A on meniscal tissue degradation and the importance of a combination with a mechanical injury (MI) was to be examined. It was shown for the first time that IL-17A has a direct degradative impact on the meniscus and that MI can alter these events through additive or even synergistic effects. The main finding was that IL-17A induced proteoglycan degradation by causing an up-regulation of sGAG release and aggrecanase activity. After stimulation of the meniscal explants with IL-17A and MI, there was an increase in sGAG release as well as fluorescence signals for NITEGE, the mRNA levels of MMP-13 and ADAMTS4, and the number of condensed cells.

Synergistic interactions between cytokines and MI had already been shown for articular cartilage and other types of cytokines, such as IL-6 and TNF-α [17,19], but for meniscal tissue and IL-17A, we showed this for the first time. In that context, our histological studies demonstrated that the NITEGE fluorescence signal was increased due to IL-17A in the superficial zone, whereas MI also caused a signal increase in deeper zones, which was further intensified by the combination of both stimuli. Sui et al. [19] found that MI potentiates the catabolic effects of TNFα and IL-6/sIL-6R in causing proteoglycan degradation in human and bovine articular cartilage and speculated that the temporal and spatial evolution of degradation suggested the importance of transport of biomolecules, which may be increased by overload injury.

We show slight increases in enzyme mRNA levels due to cytokine or injury treatment, which intensify when combined. Aggrecan cleavage is also increased, shown by staining of the cleavage product NITEGE, which intensifies when cytokine and MI are combined. As a consequence, GAG release is increased indicating proteoglycan degradation, which correlates with the effects above. However, stimulation with IL 17A alone did not result in a dose-dependent difference in the RNA expression of ADAMTS4 and MMP13 between 1 ng and 10 ng. Nevertheless, a dose-dependent effect was observed when IL 17A was combined with mechanical injury. While we cannot explain this finding at present, it suggests that distinct activation mechanisms may govern protein production under combined stimulation conditions. The mRNA increases for the enzymes might seem to be small in that context; however, it has been shown that the activity of, for example, aggrecanases can be regulated in many ways, and it remains unclear whether IL-17A uses other pathways than mRNA expression for aggrecanase activation. The review by Rose et al., “Regulation of ADAMTS Proteases”, for example, summarizes multilevel regulation of ADAMTS enzymes by transcriptional, post-transcriptional and post-translational modifications (pro-domain removal by furin, C-terminal processing), cell-surface binding, and endogenous inhibitors (TIMP-3). It explains how the activity of ADAMTS enzymes can change independently of mRNA via different ways of processing, inhibitor changes, or localization [35].

Rolauffs et al. [36] demonstrated that when articular cartilage was mechanically injured injury-related damage varied with depth due to the depth-dependent nature of ECM composition, structure, and biomechanical properties. The authors showed that superficial tissue was especially vulnerable and speculated that the biomechanical failure of the superficial zone is most likely be associated with changes in the ECM and subsequently the permeability. Taking this into consideration, a mechanically injured meniscus might also have a higher permeability for IL-17A to pass through to deeper zones; the fact that NITEGE signals increased in deeper areas of the meniscal tissue after combined injurious and cytokine treatment in our study supports this theory.

Recent insights from the epiligament theory proposed by Georgiev and colleagues [37] suggest that a specialized connective tissue layer surrounding ligaments—the epiligament—plays a critical role in healing by providing vascular, neural, and cellular support [38]. This compartment harbors fibroblast-like and progenitor cells and shows strong expression of matrix-remodeling enzymes such as MMP-2 and MMP-9 as well as angiogenic factors including VEGF [39]. Drawing an analogy to the meniscus, the vascularized perimeniscal tissue and meniscocapsular attachments may act in a comparable fashion, supplying reparative cells, vessels, and proteases to adjacent regions. Such parallels could account for the zone-dependent regenerative potential observed between the inner avascular and outer vascular meniscal zones. Further studies should explore whether epiligament-like structures exist within the perimeniscal environment and contribute to local repair, offering new perspectives for targeted meniscal regeneration strategies [10,40,41].

The findings of Kamiab et al., 2024 [42] demonstrated that serum IL-17A concentrations did not differ significantly between patients with knee osteoarthritis and healthy controls (5.52 ± 0.35 pg/mL vs. 6.20 ± 1.11 pg/mL), although a non-significant trend toward higher values was observed in the latter group. In contrast, Snelling et al. (2017) [11] identified the presence of IL-17 within synovial fluid as a pathological feature, reporting a median concentration of 7.9 pg/mL in patients with osteoarthritis. Furthermore, Chen et al., 2014a) [13] found a positive association between IL-17 levels and disease severity: serum IL-17 concentrations were significantly elevated in OA patients (n = 98) compared with healthy subjects (n = 50), and synovial fluid IL-17 levels increased progressively with Kellgren–Lawrence grade, showing a significant positive correlation with the Lequesne index (r = 0.6232). Since in vivo measurements revealed lower values than we needed to produce significant effects in vitro, we suggest that, due to the in vitro design of this study, higher concentrations of IL-17A might have been needed to elicit measurable effects on meniscal tissue within a short incubation period (72 h). It can be assumed that lower concentrations of IL-17A, as reported in vivo, may induce similar effects in vivo over a prolonged time period of weeks or even months. It is a known fact in many cytokine studies that higher cytokine levels are needed in vitro than being found in vivo. IL-17 does not seem to be an exception: the in vitro study by Sinkeviciute et al. (2020) [14] demonstrated that IL-17 concentrations of 25 ng/mL and 100 ng/mL effectively induced cartilage degradation, providing a comparable model and range of concentration to the present study [14].

The selected concentration of 10 ng IL-17A /mL was the lowest dose in vitro that showed a significant increase in sGAG release compared with the control under the given circumstances. The chosen concentration also enabled us to compare the results to existing literature data, since there was already some work on the in vitro-effect of IL-17A on articular cartilage, where IL-17A achieved pro-inflammatory effects, such as degradation of proteoglycans, at a concentration of 10 ng/mL [14,40,43]. Overall, our data support the thesis that IL-17A can participate in joint damaging, especially, as we show here, by alterations in the integrity of the meniscus.

By combining IL-17A and MI, this work was able to show a synergistic degenerative effect, which is important for the understanding of the pathogenesis of OA, especially PTOA, since the latter is the result of an injury with a subsequent pro-inflammatory tissue reaction [19,44,45]. The signal cascade triggered by IL-17A in chondrocytes of articular cartilage consists of the induction and production of IL-1β, TNFα and IL-6 [46], and the inflammatory effect of this cytokine is also triggered by the induction of chemokines such as IL-8 which leads to an aggravation of, among other things, neutrophilic granulocytes, which—again—promotes joint inflammation [47]. Together with the effects of IL-17A on meniscal tissue shown in the present study, this promotes the potential importance of IL-17A in joint degeneration and allows the conclusion that IL-17A might be a target for a therapeutic intervention in OA/PTOA]. In fact, there are already first clinical studies that evaluated the use of an IL-17 antagonist in rheumatology-related research [48,49]. The tested patients had positive outcomes concerning swelling and pain management, but only psoriatic- or rheumatoid-arthritis patients were tested; therefore, the effect on OA/PTOA still has to be determined.

On the other hand, IL-17 signaling is not strictly pathogenic by initiating harmful inflammatory responses: it also has beneficial roles in mediating tissue homeostasis, and contributes to regeneration after tissue injury including bone fracture and muscle damage (see recent review by Adamopoulos and Kuchroo [50]); therefore, the interplay between effectors and transducers that regulate the pleiotropic effects of IL-17A (and IL-17F) in tissue homeostasis and inflammation needs to be carefully examined to exploit the beneficial effects of IL-17 on one hand but inhibit its pro-inflammatory effects (as they are shown here for the meniscal tissue) on the other hand [50].

In the MI study by Hufeland et al. [26], meniscal ADAMTS4 mRNA levels were actually downregulated by injury, and the average peak stress during injurious meniscal compression was lower than in the present study. A reason for these differences might be a different quality or health status of the meniscal tissue since livestock farming was different for the animals used in the two studies: animals in the Hufeland study were from factory farming and even control tissue showed high levels of cells with condensed nuclei in histological sections and extremely high numbers of condensed and apoptotic cells after injury, whereas in the present study, animals were from species-appropriate animal husbandry with little to no damaged cells in control tissue and much less damaged cells in stimulated tissue, and therefore, there might have been still enough living cells being able to respond differently to the cytokine stimulation even after mechanical stress with an up-regulation of enzyme transcription and activity. It should also be noted that the absolute sGAG loss in the meniscal tissue is lower than in articular cartilage, which is probably due to a generally lower meniscal content of sGAGs [51]. In relation to the relative sGAG release, however, there was also a dose-dependent and significant increase, which showed that IL-17A achieved matrix-degrading effects in meniscal tissue comparable to those in articular cartilage.

Given the in vitro nature of the study, several methodological limitations must be acknowledged. Notably, bovine meniscal tissue was employed due to the restricted accessibility of healthy human tissue in a non-arthritic condition. The transferability of the data to the human system therefore remains to be clarified. Secondly, we induced a trauma by a unidirectional/axial compression, whereas the typical trauma mechanism described for meniscal injury might contain shear and traction forces as well, like the pivot-shift injury [52]. In vitro injury models including overload shear forces have been established for articular cartilage [53] and demonstrate that this type of injury adds an additional important aspect to the understanding of injury-related joint destruction. However, many of the tissue responses to both injury by shear or axial loading are related to each other and involve the same pathways or structural changes, which is why it is assumed that the data in the present study can be seen as a valid indicator for the importance of the interaction between IL-17A and mechanical injury in general.

## 5. Conclusions

Our findings emphasize an interaction between the inflammatory cytokine IL-17A signaling and mechanical stress, since IL-17A induced matrix degeneration in meniscal tissue, which, in combination with a trauma, intensified. The latter might create a post-traumatic environment that promotes meniscal degeneration and subsequently osteoarthritis progression.

## Figures and Tables

**Table 1:** Bovine primer sequencies for qRT-PCR.

Primer-Target	NCBI Identification Number	Primer Sequence (5′-3′)
GAPDH Sense	NM_001034034.2	ATCAAGAAGGTGGTGAAGCAGG
GAPDH Antisense	NM_001034034.2	TGAGTGTCGCTGTTGAAGTCG
MMP-13 Sense	NM_174389.2	TCTTGTTGCTGCCCATGAGT
MMP-13 Antisense	NM_174389.2	GGCTTTTGCCAGTGTAGGTGTA
ADAMTS4 Sense	NM_181667.1	GCGCCCGCTTCATCACTG
ADAMTS4 Antisense	NM_181667.1	TTGCCGGGGAAGGTCACG

## Data Availability

The dataset analyzed during the current study is available from the corresponding author on reasonable request.

## Abbreviations

The following abbreviations are used in this manuscript:

OA	Osteoarthritis
IL	Interleukin
MI	Mechanical injury
sGAG	Sulfated glycosaminoglycan
PTOA	Post-traumatic OA
ECM	Extracellular matrix
TNF-α	Tumor necrosis factor-α
MMP	Matrix metalloproteinase
PCR	Polymerase chain reaction
